# Quantification of global transcription patterns in prokaryotes using spotted microarrays

**DOI:** 10.1186/gb-2007-8-12-r265

**Published:** 2007-12-13

**Authors:** Ben Sidders, Mike Withers, Sharon L Kendall, Joanna Bacon, Simon J Waddell, Jason Hinds, Paul Golby, Farahnaz Movahedzadeh, Robert A Cox, Rosangela Frita, Annemieke MC ten Bokum, Lorenz Wernisch, Neil G Stoker

**Affiliations:** 1 Department of Pathology and Infectious Diseases, Royal Veterinary College, Royal College Street, London, NW1 0TU, UK; 2 School of Crystallography, Birkbeck College, London, WC1E 7HX, UK; 3 TB Research, CEPR, Health Protection Agency, Porton Down, Salisbury, SP4 0JG, UK; 4 Medical Microbiology, Division of Cellular and Molecular Medicine, St George's University of London, Cranmer Terrace, Tooting, London, SW17 0RE, UK; 5 Veterinary Laboratories Agency, Woodham Lane, New Haw, Addlestone, Surrey, KT15 3NB, UK; 6 Institute for Tuberculosis Research College of Pharmacy, University of Illinois at Chicago, Chicago, Illinois, 60612-7231, USA; 7 Division of Mycobacterial Research, National Institute for Medical Research, The Ridgeway, Mill Hill, London, NW7 1AA, UK; 8 Department of Infectious and Tropical Diseases, London School of Hygiene and Tropical Medicine, London, WC1E 7HT, UK

## Abstract

We describe an analysis, applicable to any spotted microarray dataset produced using genomic DNA as a reference, that quantifies prokaryotic levels of mRNA on a genome-wide scale. Applying this to *Mycobacterium tuberculosis*, we validate the technique, show a correlation between level of expression and biological importance, define the complement of invariant genes and analyze absolute levels of expression by functional class to develop ways of understanding an organism's biology without comparison to another growth condition.

## Abstract 2

An analysis is described, applicable to any spotted microarray dataset that is produced using genomic DNA as a reference for quantifying prokaryotic levels of mRNA on a genome-wide scale.

## Background

The biological landscape has been transformed by the sequencing of genomes, and more recently by global gene expression analyses using microarrays [1,2]. Microarrays contain DNA probes representing all coding sequences in a genome, which are either synthesized *in situ *or are spotted onto a modified glass surface [3]. Comparison of mRNA from two conditions by competitive hybridization to these probes is used to identify differentially expressed genes [1]. In the case of spotted microarrays, these are performed either with labeled cDNA prepared from separate mRNA preparations co-hybridized to the same array, or as is increasingly the case, by employing genomic DNA (gDNA) as a standard reference [4]. In the latter case, each cDNA preparation is hybridized separately alongside a gDNA reference and differential expression is determined using a ratio of ratios. The use of gDNA corrects for most spatial and spot-dependent biases inherent with microarrays, and also allows direct comparison between multiple datasets [4]. These are sometimes called type 2 experiments, with RNA:RNA hybridizations being type 1 [5]. Traditionally, microarray experiments focus almost exclusively on changes in gene expression, and in the case of a type 1 experiment this is the only possible interpretation.

Focusing on changes in expression has helped to direct us toward genes that warrant further investigation; however, it has been shown in recent meta-analyses that up-regulated genes may bear little correlation to other measures of biological importance [6-8]. One reason for this lack of correlation is that, in a traditional microarray experiment, absolute levels of mRNA are not considered; thus, no difference is reported between a gene where expression increases from 20 to 100 copies and one where it increases from 20,000 to 100,000 copies, yet the biological inference may be very different. Furthermore, all genes whose level of expression does not alter significantly between conditions are completely ignored and we do not know if they are constitutively off or on (and if so, at what level). Differential expression analysis thus provides us with an incomplete view of the transcriptome, whereas the determination of global mRNA levels could, in part, address this.

Global mRNA abundance analysis is particularly applicable in prokaryotes, where, in contrast to the situation in eukaryotes, transcription and translation are tightly coupled [9,10]. In prokaryotes, therefore, absolute mRNA levels might be expected to accurately predict levels of protein. In support of this, it has been shown in both *Escherichia coli *and *Mycobacterium smegmatis *that the most readily detectable (and hence most abundant) proteins correspond to genes with high transcript levels [11,12]. Also, in experiments where transcriptomic and proteomic data were compared, for the majority of genes, changes at the transcriptional level were mirrored at the protein level [13,14]. Furthermore, a comprehensive study of mRNA and protein levels in a sulfur-reducing bacterium identified a modest global correlation between the two but found that the majority of the variation could be attributed to errors in the protein analytical techniques, indicating the actual correlation could be much stronger [15].

Surprisingly, the study of absolute levels of mRNA on a global scale has largely been ignored, despite attempts that have been made to extract meaningful quantitative information from microarrays. These include spiking various control samples of known concentration into the hybridization mixture [16,17], and using synthesized oligos complementary to every spot on an array at a known concentration as a normaliser [18]. Another recent report described the use of the Affymetrix gene chip platform to provide a quantitative view of gene expression levels in prokaryotes [19]. These approaches are often impractical or, especially with commercial systems, prohibitively expensive. Type 2 experiments performed on spotted arrays on the other hand, which use gDNA as a reference, are already routinely being performed, require a minimal cost increase and could allow us to study the relative abundance of each mRNA species [17] in parallel with traditional fold expression analyses.

Here we have focused on the determination of genome-wide mRNA levels of *M. tuberculosis *using type 2 microarrays for which we had a large number of datasets available. We have developed and validated the approach, characterized the genes whose level of expression is the highest in the transcriptome *in vitro *and those whose level of expression remains consistently high across a variety of environmental conditions. In addition, we have coupled genome-wide levels of mRNA abundance with a functional classification system in order to develop ways of understanding an organism's biology without comparison to another growth condition.

## Results and discussion

### Calculating relative mRNA abundance

Genome-wide transcriptional analyses have until now focused almost exclusively on differential expression. Methods that have been developed to quantify absolute mRNA abundances have largely been ignored or proved impractical. However, the use of gDNA as a reference channel in traditional microarray experiments is increasingly common [4], and as this is equivalent to an equimolar concentration of all transcripts we have investigated using this as a normaliser that would allow us to generate a measure of genome wide mRNA abundances.

Initially we calculated relative mRNA abundances for *M. tuberculosis *growing aerobically in chemostat cultures as we had access to the RNA used for hybridization to the arrays, allowing us to experimentally validate our analysis. RNA and microarray procedures were carried out as described in the Materials and methods and [20]. To obtain a measure of mRNA abundance, we first removed the local background fluorescence from each probe spot on the microarray. The fluorescence intensity from the RNA channel was then normalized to that of the gDNA channel, after which the technical and biological replicates were averaged.

**Figure 1:**
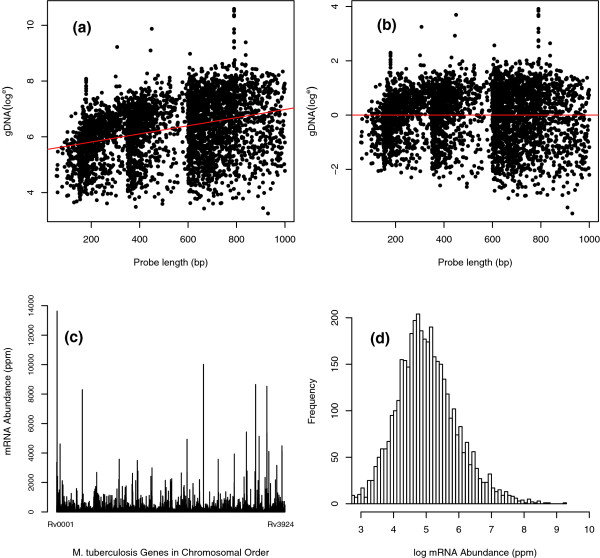
Probe length normalization and quantified gene expression levels in *M. tuberculosis*. **(a) **We found that longer probes correlated with increased fluorescent intensities, which then biased the ppm values we obtained. **(b) **We are able to remove this bias using a model of linear regression. The three distinct groupings visible in the figure are an artifact of the probe lengths targeted during their synthesis by PCR. **(c) **The level of expression for each gene in the genome, as determined by our analysis from chemostat grown wild-type *M. tuberculosis *H37Rv, is shown ordered as they appear in the chromosome. **(d) **The log frequency distribution of mRNA abundances from (c). A clear skew to the right, containing a subset of very highly expressed genes, is typical of the distributions we have found.

We then found it necessary to correct for a probe length effect present in the data. In a traditional microarray (type 1) experiment, comparisons are made between two cDNA populations hybridized to one spot. Although in most cases it is necessary to control for factors such as the spatial dependent effects of hybridization, and normalizations such as loess are routinely implemented for this purpose [21], it is not necessary to control for individual spot variations as these would be negated when calculating fold expression ratios. In our case, where we are attempting to draw comparison between signals generated from different spots on the array, we are faced with additional factors that could introduce bias to our results - those that differ between spots on the array, for example, the probe GC content and length. Using the signal reported from a gDNA hybridization, we investigated these factors. We found that the length of the probes, which ranged in length from 60 to 1,000 bp, affected the hybridization whereby longer probes report higher signal intensities (Figure 1a). We suspect this may be because larger probes are able to bind more DNA and bind it more strongly than shorter probes. We corrected for this effect using a model of linear regression (Figure 1b; and see Materials and methods).

Finally, as the sum of all fluorescence intensities is equal to the sum of all labeled mRNA, the measures of mRNA abundance were converted from unintuitive ratios to a proportional value; parts per million (ppm). Table 1 shows the mRNA abundance values for the 50 most highly expressed genes of *M. tuberculosis*. Figure 1c shows the mRNA levels, in ppm, for each gene in the *M. tuberculosis *chromosome and their log distribution is shown in Figure 1d. It is clear that the mRNA abundances, even once log transformed, are not normally distributed, which reflects the observations of others [22].

**Table 1:** The 50 most highly expressed genes *in vitro*

	Rv	Name	ppm	Function [40]
1	Rv0009	ppiA	13,634	Protein translation and modification
2	Rv2527	Rv2527	10,020	Conserved hypotheticals
3	Rv3418c	groES	8,651	Chaperones-heat shock*
4	Rv3615c	Rv3615c	8,531	Conserved hypotheticals*
5	Rv0440	groEL2	8,300	Chaperones-heat shock*
6	Rv3258c	Rv3258c	5,430	Unknown*
7	Rv3616c	Rv3616c	5,370	Conserved hypotheticals*
8	Rv3477	PE31	5,138	PE subfamily
9	Rv2244	acpM	4,935	Synthesis of fatty and mycolic acids*
10	Rv0060	Rv0060	4,616	Unknown*
11	Rv3874	Rv3874	4,490	Conserved hypotheticals
12	Rv3875	esat6	4,115	SP, L, P and A^†^
13	Rv3648c	cspA	4,101	Adaptations and atypical conditions*
14	Rv3053c	nrdH	3,934	2'-Deoxyribonucleotide metabolism
15	Rv3614c	Rv3614c	3,748	Conserved hypotheticals*
16	Rv1078	pra	3,582	Conserved hypotheticals
17	Rv2780	ald	3,577	Amino acids and amines
18	Rv1388	mIHF	3,499	Nucleoproteins*
19	Rv0003	recF	3,371	DNA R, R R and R^‡^
20	Rv3786c	Rv3786c	3,164	Unknown
21	Rv1641	infC	2,993	Protein translation and modification*
22	Rv3269	Rv3269	2,778	Chaperones-heat shock
23	Rv1398c	Rv1398c	2,777	Conserved hypotheticals
24	Rv3407	Rv3407	2,732	Conserved hypotheticals
25	Rv2245	kasA	2,693	Synthesis of fatty and mycolic acids*
26	Rv0685	tuf	2,688	Protein translation and modification*
27	Rv2145c	wag31	2,652	SP, L, P and A*^†^
28	Rv1306	atpF	2,620	ATP-proton motive force*
29	Rv0005	gyrB	2,466	DNA R, R R and R*^‡^
30	Rv1305	atpE	2,455	ATP-proton motive force*
31	Rv0016c	pbpA	2,423	Murein sacculus and peptidoglycan
32	Rv1622c	cydB	2,418	Electron transport*
33	Rv3219	whiB1	2,373	Repressors-activators
34	Rv3583c	Rv3583c	2,345	Repressors-activators
35	Rv1980c	mpt64	2,254	SP, L, P and A^†^
36	Rv1072	Rv1072	2,205	Other membrane proteins
37	Rv2457c	clpX	2,200	Proteins, peptides and glycopeptides*
38	Rv1958c	Rv1958c	2,195	Unknown
39	Rv3763	lpqH	2,180	Lipoproteins (lppA-lpr0)
40	Rv1872c	lldD2	2,149	Aerobic respiration
41	Rv3461c	rpmJ	2,133	Ribosomal protein synthesis*
42	Rv1361c	PPE19	2,127	PPE family
43	Rv0097	Rv0097	2,123	Conserved hypotheticals
44	Rv1971	Rv1971	2,034	Virulence
45	Rv3051c	nrdE	1,984	2'-Deoxyribonucleotide metabolism*
46	Rv2346c	Rv2346c	1,937	Conserved hypotheticals
47	Rv3679	Rv3679	1,931	Anions*
48	Rv1298	rpmE	1,883	Ribosomal protein synthesis*
49	Rv0108c	Rv0108c	1,837	Unknown
50	Rv2193	ctaE	1,793	Aerobic respiration*

*Essential genes (TraSH) [26,27]. ^†^Surface polysaccharides, lipopolysaccharides, proteins and antigens. ^‡^DNA replication, repair, recombination and restriction-modification.

### Validation of mRNA abundance data

**Figure 2:**
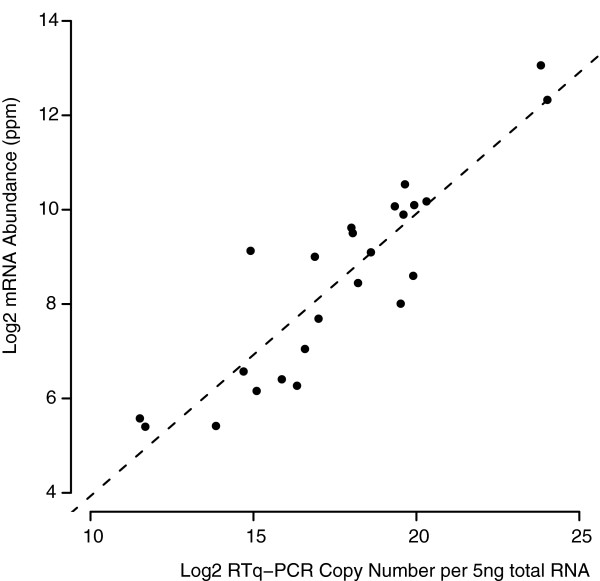
Microarray analysis validation. There is a strong correlation (0.86, Spearman's rank, *p *< 0.0001) between mRNA levels as predicted by our microarray analysis and mRNA copy number as determined by RTq-PCR.

In order to validate our estimates of abundance, we performed quantitative reverse transcriptase PCR (RTq-PCR) on a large selection of genes that we had predicted to span the mRNA abundance spectrum (*n *= 24). The RTq-PCR was carried out on a sample of the same RNA used for the microarray hybridizations. The measures of mRNA abundance as predicted from the microarray analysis show a good correlation (Spearman's rank = 0.86, *p *< 0.0001) with the absolute copy number as determined by RTq-PCR data (Figure 2).

Further validation of the method was provided by its high reproducibility when applied to data sets from independent laboratories using the same microarray designs. Correlations were determined for a variety of mRNA abundance data from both *M. tuberculosis *and the highly similar *Mycobacterium bovis *[23]. Chemostat-grown *M. tuberculosis *showed a correlation of 0.8 (*p *< 0.0001) with the homologous genes in chemostat-grown *M. bovis*. The same was true of batch-cultured *M. tuberculosis *and *M. bovis *grown in different institutions. However, there was a lower correlation of 0.5 (*p *< 0.0001) between chemostat and batch cultured *M. tuberculosis *from different laboratories, suggesting that the method of culture significantly affects the transcriptome.

### mRNA abundance, protein abundance and gene importance

**Figure 3:**
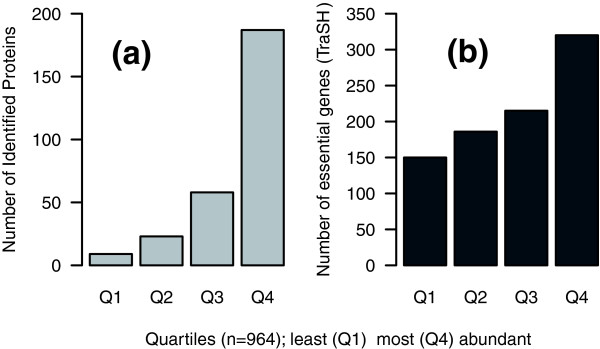
Correlations between mRNA and biological importance. **(a) **Proteins identified by two-dimensional PAGE/MS [24] correlates with the most highly expressed genes (Chi-squared test for trend in proportions = 251.9, df = 1, *p *value < 0.0001). **(b) **Similarly, there is a significant relationship between expression level and essentiality as determined by TraSH [7,26,27] (Chi-squared test for trend in proportions = 161.2, df = 1, *p *value < 0.0001).

To explore the possibility that global measures of mRNA abundance are an important indicator of prokaryotic biology, we compared our mRNA abundance data with proteome and gene essentiality data. Demonstrating a correlation between mRNA and protein levels is difficult without the availability of genome-wide measures of protein abundance. We looked instead at the list of *M. tuberculosis *proteins identified to date from two-dimensional PAGE analysis and stored in an online database [24]. As the mRNA abundances are not normally distributed, we determined the frequency of identified proteins in each quartile of the abundance distribution. Of the 283 unique proteins identified in *M. tuberculosis *cell lysates and supernatants, the majority (187 proteins, 66%) are expressed in the most abundant quartile (Figure 3a). Others have suggested that proteomic experiments have an intrinsic bias toward abundant proteins [12,25] and this would support our hypothesis that the most abundant transcripts produce high levels of protein in bacteria. In addition, our analysis makes no allowance for differential rates of translation initiation or mRNA/protein degradation, so this finding reflects how tightly coupled the systems of transcription and translation are in prokaryotes [9,10].

As there is little correlation between reports of biological importance and gene induction [6-8] we instead looked at the correlation with mRNA abundance. We compared the genome-wide values of mRNA abundance with the genes identified as being essential in *M. tuberculosis *by genome-wide transposon mutant library (TraSH) screens [7,26,27]. For our RTq-PCR validated data from *M. tuberculosis *growing under aerobic chemostat conditions we found that there is a significant relationship between expression level and essentiality on a global scale (Chi-squared test for trend in proportions = 161.2, df = 1, *p *value < 0.0001; Figure 3b). This is in contrast to the lack of correlation with fold-induction upon infection [6,7] and illustrates the potential importance of determining mRNA abundances on a global scale. The correlation may reflect our previous finding that the more highly expressed a gene, the more protein is produced. Although there are obvious examples where proteins with essential functions, such as the cell division apparatus or many enzymes, need not be (or indeed cannot be) highly expressed [28], prokaryotic cells would waste considerable energy synthesizing large amounts of proteins that do not have essential functions.

### The most highly expressed genes of *M. tuberculosis*

The genome-wide distribution of mRNA levels from *M. tuberculosis *cultured aerobically in the chemostat is typical of the distributions we have found (Figure 1d). Despite being log transformed, the distribution shows a skew to the right, suggesting the presence of a highly expressed subpopulation. As we and others have shown, the coupling of transcription and translation in prokaryotes means that there is an enormous material investment in transcribing a gene at a high level. We therefore analyzed the most abundant mRNAs in some detail, focusing on the 95th percentile of genes, that is, the 5% most abundant transcripts, *n *= 198 (Additional data file 1). Of these 198 genes, 89 (45%) were reported as essential in the TraSH experiments, which is significantly higher than the 23% of all genes that are essential (Χ^2 ^*p *< 0.001). Of the 89 essential genes, 76 (38%) were essential *in vitro*, 11 (5%) *in vivo *and 2 (1%) are essential for survival in macrophages [7,26,27].

The most highly expressed gene *in vitro *was *ppiA *(*Rv0009*), a probable peptidyl-prolyl cis-trans isomerase involved in protein folding, which makes up 13,634 ppm (which is equivalent to 1.3%) of the total mRNA population. As would be expected, many of the very abundant transcripts belong to the protein synthesis machinery, including thirty ribosomal proteins, six translation initiation factors and various components of RNA polymerase.

Several of the very abundant genes have previously been characterized as highly expressed and extensively documented as important virulence determinants of *M. tuberculosis*. In particular, some members of the *esx *gene family have been shown to be critical in infection, although dispensable *in vitro*. The paradigms for this family are *esat6 *(*Rv3875*) and *cfp10 *(*Rv3874*), whose products form a secreted complex that interacts with host cells [29]. Furthermore, they are potent immunogens with potential roles as both subunit vaccines and diagnostic agents [30,31]. The *esx *family in *M. tuberculosis *contains 23 *esat6*-like genes, with 11 *esat*/*cfp *gene pairs [32]. Including *esat6 *and *cfp10*, we identified 12 members (52%) of this family as being amongst the most highly transcribed of all genes. One such pair of genes, *esxV *(*Rv3619c*) and *esxW *(*Rv3620c*), are adjacent to, but not transcribed with, the SNM (secretion in mycobacteria) operon containing *Rv3616c *(*espA*), *Rv3615c *(*snm9*) and *Rv3614c *(*snm10*), which we also find are very highly expressed. Two groups have recently shown that the SNM system functions to export both ESAT-6 and CFP-10 [33,34].

We have also observed five highly expressed transcripts belonging to the PE/PPE family; a set of approximately 100 genes encoding proteins with proline and glutamate rich motifs that are found exclusively within the mycobacteria [35]. Some members of this family are located adjacent to *esx *genes, suggesting a functional association, and it is now known that a PE/PPE pair form a stable dimer, reminiscent of ESAT-6/CFP-10 themselves [36]. Of the five PE/PPE genes in our very abundant transcript list, two are located adjacent to highly expressed *esx *family gene pairs: *PPE18 *(*Rv1196*) with *esxKL *(*Rv1197*/*Rv1198*), and *PE19 *(*Rv1791*) with *esxMN *(*Rv1792*/*Rv1793*). The significance of linked high expression levels between *esx *and PE/PPE genes suggests a co-functionality critical to *M. tuberculosis *biology.

### Genes of unknown function

Thirty-three percent of the coding sequences in *M. tuberculosis *were classified as having no known function in a re-annotation of the genome [37]. A similar proportion (60 of 198, 30%) of the transcripts we have classified as very abundant in *M. tuberculosis *are annotated as unknown, hypothetical or conserved hypothetical proteins. The organism consumes considerable energy in their expression so it is likely they have important functions, and indeed 12 of these unknown genes are essential. Using both BLASTP and functional predictions generated with the hidden Markov model profile tool SHARKhunt [38], we were unable to ascribe any further functions to these genes with the exception of *Rv0097*, which has close homology to a taurine dioxygenase of the *Streptomyces*, *Ralstonia *and *Chromobacterium *species. It has been reported [39] that the *Rv0097 *homologue in *M. bovis *is located in an operon essential for the synthesis of the virulence associated lipids phthiocerol dimycocerosate esters (PDIMs) and could function as an α-ketoglutarate-dependent dioxygenase, the super family to which taurine dioxygenases belong.

### The invariome

Much effort goes into looking for genes whose expression is modulated in different environments. Although it is likely that most, if not all, genes are regulated to some extent, using the analysis described here it is possible to search for genes whose expression does not change significantly, which we term 'invariant'. These may represent genes whose functions are so important that they cannot be switched off. To identify these invariant genes, we compared the mRNA abundance in a total of six data sets including various growth conditions, such as chemostat, batch culture, low oxygen and growth in macrophages. We focused on genes that were within the 85th percentile and found that 133 genes were consistently highly expressed across all of the conditions tested (Table 2).

**Table 2:** The 133 genes of the 'abundant invariome'

	Rv	Name	Avg ppm	Stdev	Essential
1	Rv3874	lhp	5,414	3,950	-
2	Rv3418c	groES	5,189	2,593	*In vitro*
3	Rv0440	groEL2	4,438	2,385	*In vitro*
4	Rv3615c	Rv3615c	3,887	2,539	*In vivo*
5	Rv0009	ppiA	3,460	4,587	-
6	Rv3616c	Rv3616c	2,619	1,457	*In vivo*
7	Rv3477	PE31	2,537	1,553	-
8	Rv2244	acpM	2,475	1,304	*In vitro*
9	Rv3875	esat6	2,472	1,229	-
10	Rv1398c	Rv1398c	2,449	1,311	-
11	Rv3648c	cspA	2,372	1,149	*In vitro*
12	Rv2245	kasA	2,236	481	*In vitro*
13	Rv3614c	Rv3614c	2,232	847	*In vivo*
14	Rv1307	atpH	2,151	1,195	*In vitro*
15	Rv0667	rpoB	2,105	563	*In vitro*
16	Rv1388	mihF	2,103	1,013	*In vitro*
17	Rv0685	tuf	2,100	792	*In vitro*
18	Rv3583c	Rv3583c	2,096	2,027	-
19	Rv3053c	nrdH	1,930	1,339	-
20	Rv1133c	metE	1,915	323	*In vitro*
21	Rv1072	Rv1072	1,897	560	-
22	Rv1872c	lldD2	1,817	574	-
23	Rv3461c	rpmJ	1,814	795	*In vitro*
24	Rv2457c	clpX	1,790	343	*In vitro*
25	Rv0700	rpsJ	1,693	1,148	*In vitro*
26	Rv1078	pra	1,643	971	-
27	Rv1298	rpmE	1,529	543	*In vitro*
28	Rv2840c	Rv2840c	1,495	566	-
29	Rv1630	rpsA	1,491	439	*In vitro*
30	Rv0046c	ino1	1,488	620	-
31	Rv1886c	fbpB	1,464	1,168	-
32	Rv2196	qcrB	1,455	472	*In vitro*
33	Rv3443c	rplM	1,447	300	*In vitro*
34	Rv0701	rplC	1,421	880	*In vitro*
35	Rv0682	rpsL	1,419	576	*In vitro*
36	Rv3219	whiB1	1,384	795	-
37	Rv0702	rplD	1,364	619	*In vitro*
38	Rv0289	Rv0289	1,351	845	*In vitro*
39	Rv2200c	ctaC	1,332	1,406	*In vitro*
40	Rv1980c	mpt64	1,316	629	-
41	Rv1306	atpF	1,246	695	*In vitro*
42	Rv2193	ctaE	1,217	334	*In vitro*
43	Rv1310	atpD	1,184	412	*In vitro*
44	Rv1174c	Rv1174c	1,165	424	-
45	Rv1308	atpA	1,148	349	*In vitro*
46	Rv3051c	nrdE	1,123	578	*In vitro*
47	Rv1305	atpE	1,086	696	*In vitro*
48	Rv0683	rpsG	1,080	541	*In vitro*
49	Rv1297	rho	1,074	281	*In vitro*
50	Rv2461c	clpP1	1,028	346	-
51	Rv0655	mkl	1,024	385	*In vivo*
52	Rv3052c	nrdI	1,018	551	-
53	Rv3801c	fadD32	1,015	209	*In vitro*
54	Rv0005	gyrB	1,011	684	*In vitro*
55	Rv0704	rplB	1,011	528	*In vitro*
56	Rv3412	Rv3412	1,002	141	-
57	Rv0250c	Rv0250c	995	439	-
58	Rv2460c	clpP2	991	393	*In vitro*
59	Rv2204c	Rv2204c	963	277	-
60	Rv3478	PPE60	957	360	-
61	Rv0703	rplW	956	556	*In vitro*
62	Rv2094c	tatA	949	241	-
63	Rv1303	Rv1303	939	637	*In vitro*
64	Rv3456c	rplQ	937	297	-
65	Rv0719	rplF	925	406	*In vitro*
66	Rv0684	fusA1	923	367	*In vitro*
67	Rv2347c	Rv2347c	920	447	-
68	Rv0715	rplX	906	342	*In vitro*
69	Rv1197	Rv1197	906	440	-
70	Rv1479	moxR1	898	162	*In vitro*
71	Rv0718	rpsH	886	243	*In vitro*
72	Rv3460c	rpsM	882	373	-
73	Rv2194	qcrC	870	194	*In vitro*
74	Rv2195	qcrA	868	189	*In vitro*
75	Rv0860	fadB	863	420	-
76	Rv1309	atpG	861	401	*In vitro*
77	Rv0243	fadA2	859	231	-
78	Rv3248c	sahH	850	243	*In vitro*
79	Rv0020c	TB39.8	850	224	-
80	Rv3584	lpqE	845	246	-
81	Rv1793	Rv1793	836	309	-
82	Rv3620c	Rv3620c	827	375	-
83	Rv1410c	Rv1410c	815	188	*In vivo*
84	Rv3459c	rpsK	809	206	*In vitro*
85	Rv0483	lprQ	805	341	-
86	Rv3043c	ctaD	803	221	*In vitro*
87	Rv3029c	fixA	801	232	*In vitro*
88	Rv2868c	gcpE	799	384	-
89	Rv1304	atpB	796	203	*In vivo*
90	Rv1642	rpmI	784	388	-
91	Rv1794	Rv1794	781	254	-
92	Rv0288	cfp7	781	286	-
93	Rv3810	pirG	778	131	*In vivo*
94	Rv1543	Rv1543	770	222	-
95	Rv3680	Rv3680	765	294	-
96	Rv3457c	rpoA	760	293	*In vitro*
97	Rv3045	adhC	756	272	-
98	Rv1792	Rv1792	753	326	-
99	Rv2969c	Rv2969c	738	141	*In vitro*
100	Rv1177	fdxC	736	303	*In vitro*
101	Rv3867	Rv3867	735	200	-
102	Rv1038c	Rv1038c	724	391	-
103	Rv2890c	rpsB	715	120	*In vitro*
104	Rv3224	Rv3224	709	303	-
105	Rv3458c	rpsD	707	208	*In vitro*
106	Rv2785c	rpsO	690	316	-
107	Rv2986c	hupB	687	255	*In vitro*
108	Rv0174	mce1F	683	211	-
109	Rv3211	rhlE	676	251	-
110	Rv1436	gap	673	234	*In vitro*
111	Rv0351	grpE	672	282	*In vitro*
112	Rv2764c	thyA	667	239	-
113	Rv1311	atpC	660	160	*In vitro*
114	Rv0432	sodC	657	177	-
115	Rv1791	PE19	655	232	-
116	Rv0932c	pstS2	652	249	-
117	Rv2971	Rv2971	645	188	*In vitro*
118	Rv1300	hemK	644	220	*In vitro*
119	Rv2703	sigA	643	114	*In vitro*
120	Rv2203	Rv2203	633	196	-
121	Rv0423c	thiC	614	149	*In vitro*
122	Rv2587c	secD	602	267	-
123	Rv1887	Rv1887	601	148	-
124	Rv0313	Rv0313	588	136	-
125	Rv0502	Rv0502	559	147	-
126	Rv3841	bfrB	542	107	-
127	Rv2115c	Rv2115c	529	106	-
128	Rv3587c	Rv3587c	526	68	-
129	Rv2110c	prcB	516	151	*In vitro*
130	Rv1987	Rv1987	495	136	-
131	Rv2454c	Rv2454c	489	55	-
132	Rv0125	pepA	483	80	-
133	Rv1324	Rv1324	474	152	-

Using our measure of absolute mRNA levels, we have been able to identify the genes whose level of expression remains consistently high across a variety of growth conditions. These genes remain amongst the top 15% most highly expressed genes across all of the conditions tested. We have termed the genes whose level of expression does not vary greatly as invariant, and, therefore, the subset of genes included in this table is dubbed the 'abundant invariome'.

Notable members of this abundant invariome include several *esx*-related genes (*esat6*, *cfp10 *and the SNM secretion system), the paired PE31 and PPE60, *groEL2 *(the 65 kDa antigen) and the ATP synthase operon (*atpA-atpH*). Compared to a global 23% of genes that are essential, 53% (70 of 133, Χ^2 ^*p *< 0.001) of the genes in the abundant invariome are essential for either *in vitro *growth or survival in mice or macrophages [7,26,27]. Twenty-two of our abundant invariome genes have no known function, two of which (*Rv0289 *and *Rv1303*) are essential. These and other members of the abundant invariome are primary candidates for future functional studies that may elucidate key mycobacterial biology.

As well as an obvious hypothesis generation role, such invariant gene analyses might have uses, for example, in identifying strong antigens, constitutive promoters and stable housekeeping genes expressed in all environments. Furthermore, as significant proportions of the abundant invariome are essential, this analysis also has the potential to identify drug targets or candidate virulence factors in prokaryotes even if no other biological information is known.

### Highly transcribed functional categories

The advent of systems biology is encouraging the development of techniques that reveal more holistic information about biological systems. We have therefore combined our measures of *M. tuberculosis *mRNA abundance with a non-overlapping classification system based on known or predicted functions [40,41]. Not only does this accord with the aims of systems biology, it would also remove the need to routinely compare expression profiles to that of artificial laboratory conditions and could, therefore, be more biologically meaningful.

Using this analysis to study the transcriptome of *M. tuberculosis *in a disease relevant [42] low oxygen state (chemostat grown at a dissolved oxygen tension (DOT) of 0.2% [20]) reveals that, at the broadest scope of the classification system, 29% of the mRNA in the transcriptome codes for proteins involved in small molecule metabolism, 19% for macromolecule metabolism, 7% for 'other' functions (virulence, and so on) and 7% for cellular processes. In addition, 38% of the *in vitro *low oxygen transcriptome codes for proteins of unknown function, illustrating how little of mycobacterial biology has been characterized to date.

To determine which functional classes, at all levels of the classification system, were significantly over- or under-represented in the transcriptome, we chose, for comparison, three different robust and nonparametric approaches: robust linear modeling, bootstrap-*t *using the Q statistic of Davison and Hinkley [43], and a bootstrap-*t *using trimmed means and winsorized variances [44]. We removed all classes with fewer than four data points to be able to obtain variance estimates after trimming. As an example of how this might be used, we again focused on the low oxygen transcriptome. In this example, many of the functional classes that we have shown to be significantly over-represented within the transcriptome by all three tests reflect the growth rate in the chemostat (maintained at a 24 hour doubling time [20]), including the protein translation machinery, the chaperones, the RNA and DNA synthesis mechanisms, and the ribosomal proteins (Additional data file 2). Also abundant are classes involved with energy metabolism, including ATP synthesis and the TCA cycle, as well as macromolecule synthesis, including the fatty and mycolic acid anabolic pathways.

Using either of the bootstrap-*t *methods appears to be too stringent to reveal classes that we would immediately recognize as reflecting the adaptation to low oxygen. However, the classes deemed significant by the robust linear modeling method include the glyoxylate shunt enzymes, which are essential *in vivo *for the anaplerosis of acetyl-CoA when growing on fatty acids [45,46], and the oxidative electron transport system known to operate under reduced oxygen tensions in mycobacteria [47].

Our functional analyses are only preliminary; we are limited by the lack of a comprehensive bacterial gene ontology. However, we suggest that this is a biologically relevant approach that could be expanded and used to identify the key cellular and metabolic processes required by an organism in a particular growth condition. It will link well with other systems biology analyses to produce useful insights into bacterial physiological states and, for example, could be used to determine the processes, rather than the components, required for infection and latency in *M. tuberculosis*.

## Conclusion

We have developed a method of microarray analysis that quantifies levels of mRNA on a genome-wide scale. Our method of analysis can be applied to any spotted microarray data set produced using gDNA as a reference channel. Applying this analysis to the prokaryote *M. tuberculosis*, we have identified the most highly expressed genes and note correlations with gene essentiality as well as with a basic measure of protein abundance. We have also been able to define the subset of genes that are invariantly highly expressed and find that more than half are essential for growth *in vitro *or survival *in vivo*. In addition, we are also able to produce a functionally organized holistic view of the transcriptome. Alongside traditional changes in expression, mRNA abundance analysis can, therefore, greatly enhance the utility of microarray data and has numerous additional uses that will aid genetic research into prokaryotic organisms.

## Materials and methods

### Microarrays

Six microarray datasets have been used in this study (Table 3). The microarrays used for hybridization were the BμG@S TB version 1 arrays (Array Express accession: A-BUGS-1) [48], for data sets 1 to 3, containing 3,924 spotted PCR products and TB version 2 arrays (A-BUGS-23) [48], for data sets 4 to 6, containing 4,410 spotted PCR products, including additional open reading frames from *M. bovis *strain AF2122/97. Fully annotated microarray data have been deposited in BμG@Sbase (accession number E-BUGS-60) [49] and also ArrayExpress (accession number E-BUGS-60).

**Table 3:** Microarray datasets used in this study

	Description	Origin	Reference	Data storage
1	Wild-type Mtb H37Rv aerobic chemostat	CAMR, UK	[20]	BμG@Sbase: E-BUGS-60
2	Wild-type Mtb H37Rv low oxygen chemostat - 0.2% DOT	CAMR, UK	[20]	BμG@Sbase: E-BUGS-60
3	Wild-type Mtb H37Rv aerobic rolling batch culture	RVC, UK	Unpublished	BμG@Sbase: E-BUGS-60
4	Wild-type Mbovis AF2122/97 aerobic chemostat	VLA, UK	Unpublished	BμG@Sbase: E-BUGS-60
5	Wild-type Mbovis AF2122/97 aerobic rolling batch culture	VLA, UK	Unpublished	BμG@Sbase: E-BUGS-60
6	Wild-type Mtb H37Rv harvested from macrophages	SGUL, UK	Unpublished	BμG@Sbase: E-BUGS-60

### Bacterial culture

Batch cultures of *M. tuberculosis *and *M. bovis *were grown in 100 ml Middlebrook 7H9 (Becton, Dickinson and Co. Franklin Lakes, NJ, USA) supplemented with 10% Middlebrook OADC (Becton, Dickinson and Co.) in 1 liter capacity flasks that were continuously rolled at 37°C. Chemostat cultures, RNA extraction and microarray hybridizations were performed as detailed in Bacon *et al*. 2004 [20]. Briefly, 500 ml cultures were grown to steady state conditions in 1 liter chemostat fermentation vessels maintained at 37°C, with a pH of 6.9 and a generation time of 24 hours. Aerobic cultures were kept at a DOT of 10% whilst low oxygen cultures were maintained at 0.2% DOT.

### RNA and genomic DNA extraction

Aliquots of the bacterial cultures (10 ml) were sampled directly into four volumes of a guanadinium thiocyanate based stop solution. After centrifugation and resuspension in either Trizol (Invitrogen, Carlsbad, California, USA) or additional stop solution, cells were lysed using a Ribolyser (Hybaid, Teddington, England) or Precellys 24 (Bertin Technologies, Montigny-le-Bretonneux, France) and RNA was extracted using a chloroform precipitation followed by purification with the RNeasy Mini Kit (Qiagen, Hilden, Germany). RNA was then treated with deoxyribonuclease (DNase 1 amplification grade, Life Technologies Inc/Invitrogen, Carlsbad, California, USA). Genomic DNA was isolated from pellets of stationary phase mycobacterial cultures following previously described procedures [50].

### RNA/DNA labeling and hybridization

For the majority of the datasets RNA was extracted from four independent biological replicates and each was labeled in triplicate using 8 μg total RNA as a template for reverse transcriptase (Superscript II RNAse H, Life Technologies Inc.) in the presence of random primers (Invitrogen, Carlsbad, California, USA) and Cy5 labeled dCTP. Genomic DNA (1 μg) was used as a template for DNA polymerase (Klenow, Invitrogen, Carlsbad, California, USA) in the presence of random primers and Cy3 labeled dCTP.

Purified Cy3/Cy5 labeled DNA were combined and added to the array underneath a cover slip before being sealed in a hybridization chamber and submerged in a water bath at 65°C for 16-20 hours. Scanning was performed with a dual laser scanner (Affymetrix 428, MWG Biotech, Ebersberg, Germany) at a gain below saturation of the most intense spots. Both spot and local background levels were quantified from the resulting images using ImaGene 4.0 (MWG Biotech).

### mRNA abundance calculation

The Perl computing language and the R statistical environment [51] were used to perform all data and statistical analysis. The YASMA [52] microarray analysis package for R was used to input and structure the raw data.

Initially, all control spots on the array were removed from the dataset, including all representing ribosomal RNA. The local background noise, as determined by the image quantification software, was subtracted from each spot. No data values were excluded from this study as we reasoned weak signals (after background subtraction) were reflective of low abundance transcripts.

For each spot *i *on the array the fluorescent intensity from the cDNA (RNA) channel was normalized by simple division to the fluorescent intensity of the gDNA channel:

Normalized intensity (R_*i*_) = cDNA_*i*_/gDNA_*i*_

The correlation between hybridization replicates within each dataset was confirmed to ensure there were no extreme outliers. Technical and biological replicates were then averaged to provide a single normalized intensity value for each gene on the array.

To account for the observed probe length bias (see Results and discussion), signal intensity was normalized to probe length using a model of linear regression of log intensity on probe length (Figure 1a,b):

Probe normalized intensity (log^*e *^Rn_*i*_) = log^*e *^R_*i *_- (intercept + slope × probe length_*i*_)

The corrected Rn_*i *_values were converted back to a raw scale and for ease of understanding are depicted as a proportional value, expressed in ppm, based on the assumption that the sum of all intensity values represents the sum of the transcript (mRNA) population within the sample:

ppm = (Rn_*i*_/∑_*i*-*ith *_Rn) × 10^6^

### RTq-PCR

To assess if the measures of transcript abundance from the array analysis truly reflect that of the RNA sample we carried out RTq-PCR on 24 genes predicted to span the spectrum of mRNA abundances as determined by the mRNA abundance analysis. The RNA samples used were those extracted and used for the microarray hybridizations in data set 1 [20]. Total RNA (400 ng) was reverse transcribed using Superscript Reverse Transcriptase III (Invitrogen) according to the manufacturer's instructions. cDNA (5 ng) was subsequently used for RTq-PCR using the DyNAmo SYBR green qPCR kit (Finnzymes, Espoo, Finland), according to the manufacturer's instructions, in a DNA Engine Opticon 2 thermal cycler (MJ Research, Waltham, MA, USA). For each reaction a set of gDNA standards of known copy number were used to produce a standard curve from which a copy number could accurately be determined. The data were analyzed using Opticon Monitor v2.0.

### Functional category analysis

The genes of *M. tuberculosis *were grouped based on a Riley-like classification system obtained from the Sanger Institute [40]. The classification system is non-overlapping and hierarchical, and thus has six highest level functional categories: small-molecule metabolism, macromolecule metabolism, cell processes, other, conserved hypothetical and unknowns, each of which splits into more specific subcategories. In total, 3,925 genes are classified in this system.

We were looking to compare the level of expression in each functional class with that in other classes to discover which classes might be over- or under-represented within the transcriptome. We used ANOVA based approaches to detect functional classes that show significant over- or under-representation compared to the rest. We estimated location parameters for the log ppm values for each class and assessed the statistical significance of the contrast of a location parameter for a particular class and the average of location parameters for all the other classes. As seen in Figure 1d, the mRNA abundances are not normally distributed even after log-transformation, so we could not assume that the distribution of abundances within each class would be. Moreover, the variances changed considerably between functional classes. We therefore chose three different robust and nonparametric approaches to estimate the location parameters and to establish significance of contrasts: robust linear modeling; a bootstrap-*t *using the Q statistic of Davison and Hinkley [43]; and a bootstrap-*t *using trimmed means and winsorized variances [44]. We removed all classes with fewer than four data points to be able to obtain variance estimates after trimming.

For the robust linear model we used the rlm function from the R package MASS [53], with Huber's Psi function and default settings. In the bootstrap-*t *with Q values as pivot we trimmed points with their robustly estimated residues in the top 20% and bottom 20% quantiles before performing the bootstrap analysis to protect the estimations against outliers. For the second bootstrap-*t *we used trimmed means with 20% of the lower and 20% of the upper quantiles removed, corresponding to 20% winsorized variances for the pivot. Resampling was done within each functional class, since we take them as fixed effects. We collected 10,000 bootstrap samples to allow for multiple testing correction. The method of Benjamini and Yekutieli [54] was used to calculate a false discovery rate allowing for dependencies between functional classes.

The results from all tests are provided as supporting information (Additional data file 2). Classes were considered significantly different from others if the adjusted *p *value was less than 0.05.

## Abbreviations

DOT, dissolved oxygen tension; gDNA, genomic DNA; ppm, parts per million; RTq-PCR, quantitative reverse transcriptase PCR.

## Authors' contributions

BS, LW and NS designed the research; BS, MW and LW performed the research; SLK, RAC, RF, AMCtB, SW, JH, PG, FM, and JB contributed reagents or data to this study; BS, LW and NS wrote the paper.

## Additional data files

The following additional data are available with the online version of this paper. Additional data file 1 is a table listing the 198 genes of the 95th percentile; the very abundant transcripts in *M. tuberculosis*. Additional data file 2 is a table that includes the quantified level of each functional category and details of those deemed significantly more or less abundant in the low oxygen transcriptome, including data from the three approaches to assess significance.

## Supplementary Material

Additional data file 1 The 198 genes of the 95th percentile; the very abundant transcripts in *M. tuberculosis*. Click here for file

Additional data file 2 The quantified level of each functional category and details of those deemed significantly more or less abundant in the low oxygen transcriptome, including data from the three approaches to assess significance. Click here for file

## References

[B1] Schena M, Shalon D, Davis RW, Brown PO (1995). Quantitative monitoring of gene expression patterns with a complementary DNA microarray.. Science.

[B2] Conway T, Schoolnik GK (2003). Microarray expression profiling: capturing a genome-wide portrait of the transcriptome.. Mol Microbiol.

[B3] Heller MJ (2002). DNA microarray technology: devices, systems, and applications.. Annu Rev Biomed Eng.

[B4] Talaat AM, Howard ST, Hale W, Lyons R, Garner H, Johnston SA (2002). Genomic DNA standards for gene expression profiling in *Mycobacterium tuberculosis*.. Nucleic Acids Res.

[B5] Sidders B, Stoker NG, Hacker J, Dobrindt U (2006). Transcriptome analysis: towards a comprehensive understanding of global transcription activity.. Pathogenomics: Genome Analysis of Pathogenic Microbes.

[B6] Kendall SL, Rison SC, Movahedzadeh F, Frita R, Stoker NG (2004). What do microarrays really tell us about *M. tuberculosis*?. Trends Microbiol.

[B7] Rengarajan J, Bloom BR, Rubin EJ (2005). Genome-wide requirements for *Mycobacterium tuberculosis *adaptation and survival in macrophages.. Proc Natl Acad Sci USA.

[B8] Miklos GL, Maleszka R (2004). Microarray reality checks in the context of a complex disease.. Nat Biotechnol.

[B9] Miller OL, Hamkalo BA, Thomas CA (1970). Visualization of bacterial genes in action.. Science.

[B10] Cox RA (2007). A scheme for the analysis of microarray measurement based on a quantitative theoretical framework for bacterial cell growth: application to studies of *Mycobacterium tuberculosis*.. Microbiology.

[B11] Corbin RW, Paliy O, Yang F, Shabanowitz J, Platt M, Lyons CE, Root K, McAuliffe J, Jordan MI, Kustu S (2003). Toward a protein profile of *Escherichia coli*: comparison to its transcription profile.. Proc Natl Acad Sci USA.

[B12] Wang R, Prince JT, Marcotte EM (2005). Mass spectrometry of the *M. smegmatis *proteome: Protein expression levels correlate with function, operons, and codon bias.. Genome Res.

[B13] Eymann C, Homuth G, Scharf C, Hecker M (2002). *Bacillus subtilis *functional genomics: global characterization of the stringent response by proteome and transcriptome analysis.. J Bacteriol.

[B14] Yoshida K-i, Kobayashi K, Miwa Y, Kang C-M, Matsunaga M, Yamaguchi H, Tojo S, Yamamoto M, Nishi R, Ogasawara N (2001). Combined transcriptome and proteome analysis as a powerful approach to study genes under glucose repression in *Bacillus subtilis*.. Nucleic Acids Res.

[B15] Nie L, Wu G, Zhang W (2006). Correlation between mRNA and protein abundance in *Desulfovibrio vulgaris*: a multiple regression to identify sources of variations.. Biochem Biophys Res Commun.

[B16] Caldwell R, Sapolsky R, Weyler W, Maile RR, Causey SC, Ferrari E (2001). Correlation between *Bacillus subtilis *scoC phenotype and gene expression determined using microarrays for transcriptome analysis.. J Bacteriol.

[B17] Bernstein JA, Khodursky AB, Lin PH, Lin-Chao S, Cohen SN (2002). Global analysis of mRNA decay and abundance in *Escherichia coli *at single-gene resolution using two-color fluorescent DNA microarrays.. Proc Natl Acad Sci USA.

[B18] Dudley AM, Aach J, Steffen MA, Church GM (2002). Measuring absolute expression with microarrays with a calibrated reference sample and an extended signal intensity range.. Proc Natl Acad Sci USA.

[B19] Fu L, Fu-Liu C (2007). The gene expression data of *Mycobacterium tuberculosis *based on Affymetrix gene chips provide insight into regulatory and hypothetical genes.. BMC Microbiology.

[B20] Bacon J, James BW, Wernisch L, Williams A, Morley KA, Hatch GJ, Mangan JA, Hinds J, Stoker NG, Butcher PD (2004). The influence of reduced oxygen availability on pathogenicity and gene expression in *Mycobacterium tuberculosis*.. Tuberculosis.

[B21] Smyth GK, Speed T (2003). Normalization of cDNA microarray data.. Methods.

[B22] Danchin A, Sekowska A (2000). Expression profiling in reference bacteria: dreams and reality.. Genome Biol.

[B23] Garnier T, Eiglmeier K, Camus J-C, Medina N, Mansoor H, Pryor M, Duthoy S, Grondin S, Lacroix C, Monsempe C (2003). The complete genome sequence of *Mycobacterium bovis*.. Proc Natl Acad Sci USA.

[B24] Mollenkopf H-J, Jungblut PR, Raupach B, Mattow J, Lamer S, Zimny-Arndt U, Schaible UE, Kaufmann SHE (1999). A dynamic two-dimensional polyacrylamide gel electrophoresis database: The mycobacterial proteome via Internet.. Electrophoresis.

[B25] Liu H, Sadygov RG, Yates JR (2004). A model for random sampling and estimation of relative protein abundance in shotgun proteomics.. Anal Chem.

[B26] Sassetti CM, Boyd DH, Rubin EJ (2003). Genes required for mycobacterial growth defined by high density mutagenesis.. Mol Microbiol.

[B27] Sassetti CM, Rubin EJ (2003). Genetic requirements for mycobacterial survival during infection.. Proc Natl Acad Sci USA.

[B28] Stoker N, Broome-Smith J, Edelman A, Spratt B (1983). Organization and subcloning of the dacA-rodA-pbpA cluster of cell shape genes in *Escherichia coli*.. J Bacteriol.

[B29] Renshaw PS, Panagiotidou P, Whelan A, Gordon SV, Hewinson RG, Williamson RA, Carr MD (2002). Conclusive evidence that the major T-cell antigens of the *Mycobacterium tuberculosis *complex ESAT-6 and CFP-10 form a tight, 1:1 complex and characterization of the structural properties of ESAT-6, CFP-10, and the ESAT-6-CFP-10 complex. Implications for pathogenesis and virulence.. J Biol Chem.

[B30] Sorensen A, Nagai S, Houen G, Andersen P, Andersen A (1995). Purification and characterization of a low-molecular-mass T-cell antigen secreted by *Mycobacterium tuberculosis*.. Infect Immun.

[B31] Pym AS, Brodin P, Majlessi L, Brosch R, Demangel C, Williams A, Griffiths KE, Marchal G, Leclerc C, Cole ST (2003). Recombinant BCG exporting ESAT-6 confers enhanced protection against tuberculosis.. Nat Med.

[B32] Brodin P, Rosenkrands I, Andersen P, Cole ST, Brosch R (2004). ESAT-6 proteins: protective antigens and virulence factors?. Trends Microbiol.

[B33] Fortune SM, Jaeger A, Sarracino DA, Chase MR, Sassetti CM, Sherman DR, Bloom BR, Rubin EJ (2005). Mutually dependent secretion of proteins required for mycobacterial virulence.. Proc Natl Acad Sci USA.

[B34] Macgurn JA, Raghavan S, Stanley SA, Cox JS (2005). A non-RD1 gene cluster is required for Snm secretion in *Mycobacterium tuberculosis*.. Mol Microbiol.

[B35] Brennan MJ, Delogu G (2002). The PE multigene family: a 'molecular mantra' for mycobacteria.. Trends Microbiol.

[B36] Strong M, Sawaya MR, Wang S, Phillips M, Cascio D, Eisenberg D (2006). Toward the structural genomics of complexes: Crystal structure of a PE/PPE protein complex from *Mycobacterium tuberculosis*.. Proc Natl Acad Sci USA.

[B37] Camus J-C, Pryor MJ, Medigue C, Cole ST (2002). Re-annotation of the genome sequence of *Mycobacterium tuberculosis *H37Rv.. Microbiology.

[B38] Pinney JW, Shirley MW, McConkey GA, Westhead DR (2005). metaSHARK: software for automated metabolic network prediction from DNA sequence and its application to the genomes of *Plasmodium falciparum *and *Eimeria tenella*.. Nucleic Acids Res.

[B39] Hotter GS, Wards BJ, Mouat P, Besra GS, Gomes J, Singh M, Bassett S, Kawakami P, Wheeler PR, de Lisle GW (2005). Transposon mutagenesis of Mb0100 at the ppe1-nrp locus in *Mycobacterium bovis *disrupts phthiocerol dimycocerosate (PDIM) and glycosylphenol-PDIM biosynthesis, producing an avirulent strain with vaccine properties at least equal to those of *M. bovis *BCG.. J Bacteriol.

[B40] Sanger Institute M. tuberculosis H37Rv Functional Classification. http://www.sanger.ac.uk/Projects/M_tuberculosis/Gene_list/.

[B41] Cole ST, Brosch R, Parkhill J, Garnier T, Churcher C, Harris D, Gordon SV, Eiglmeier K, Gas S, Barry CE (1998). Deciphering the biology of *Mycobacterium tuberculosis *from the complete genome sequence.. Nature.

[B42] Dannenberg AM (1994). Roles of cytotoxic delayed-type hypersensitivity and macrophage-activating cell-mediated immunity in the pathogenesis of tuberculosis.. Immunobiology.

[B43] Davison A, Hinkley D (1997). Bootstrap Methods and their Application.

[B44] Lunneborg C (2000). Data Analysis by Resampling: Concepts and Applications.

[B45] McKinney JD, zu Bentrup KH, Munoz-Elias EJ, Miczak A, Chen B, Chan W-T, Swenson D, Sacchettini JC, Jacobs WR, Russell DG (2000). Persistence of *Mycobacterium tuberculosis *in macrophages and mice requires the glyoxylate shunt enzyme isocitrate lyase.. Nature.

[B46] Munoz-Elias EJ, McKinney JD (2005). *Mycobacterium tuberculosis *isocitrate lyases 1 and 2 are jointly required for *in vivo *growth and virulence.. Nat Med.

[B47] Kana BD, Weinstein EA, Avarbock D, Dawes SS, Rubin H, Mizrahi V (2001). Characterization of the cydAB-Encoded Cytochrome bd oxidase from *Mycobacterium smegmatis*.. J Bacteriol.

[B48] The Multi-Collaborative Microbial Pathogen Microarray Facility: BuG@S. http://bugs.sghms.ac.uk/.

[B49] Microarray Data Storage. http://bugs.sgul.ac.uk/E-BUGS-60.

[B50] Belisle JT, Sonnenberg MG (1998). Isolation of genomic DNA from mycobacteria.. Methods Mol Biol.

[B51] The Comprehensive R Archive Network. http://cran.r-project.org/.

[B52] Wernisch L, Kendall SL, Soneji S, Wietzorrek A, Parish T, Hinds J, Butcher PD, Stoker NG (2003). Analysis of whole-genome microarray replicates using mixed models.. Bioinformatics.

[B53] Venables W, Ripley B (2002). Modern Applied Statistics with S.

[B54] Benjamini Y, Yekutieli D (2001). The control of the false discovery rate in multiple testing under dependency.. Ann Stat.

